# Supplementary material for: Transcriptomic Analysis Reveals Candidate Genes in Response to Sorghum Mosaic Virus and Salicylic Acid in Sugarcane
Source: Plants (Basel). 2024 Jan 14;13(2):234. doi: 10.3390/plants13020234 (PMC10819896; doi:10.3390/plants13020234)
Supplement: Supplementary file 1 [file plants-13-00234-s001.zip › plants-2693948-supplementary.pdf]

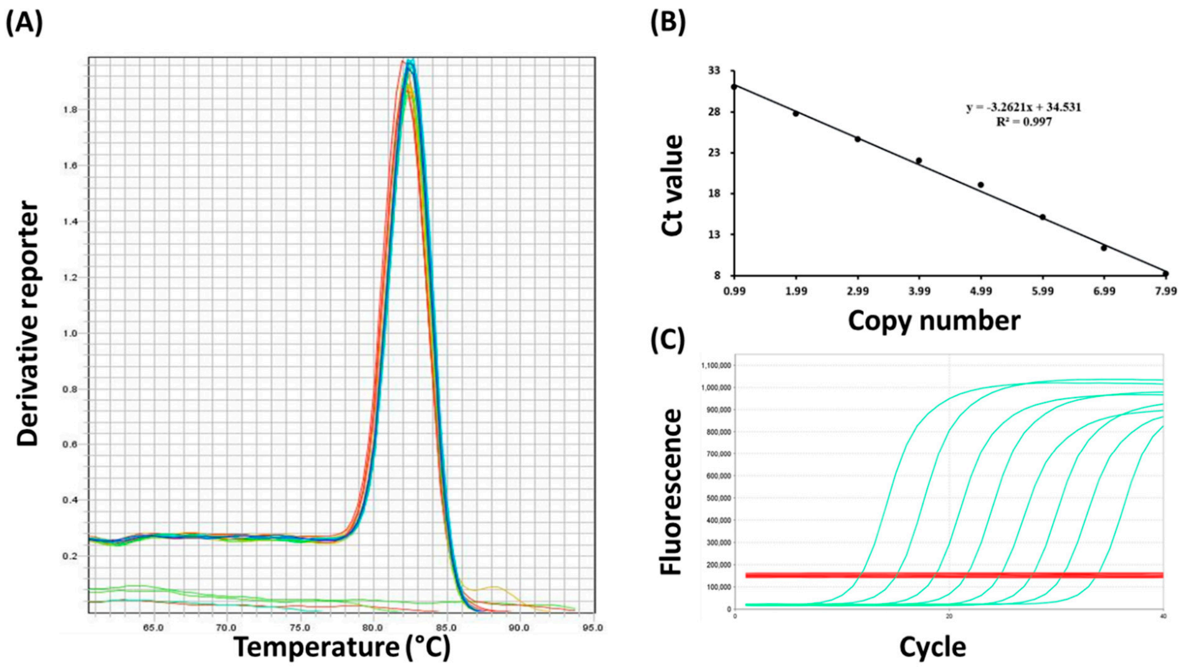

Figure S1. (**A**) Melt, (**B**) standard, and (**C**) amplification curves for determination of SrMV contents in sugarcane leaves.

Table S1. Primers used for qRT-PCR to detect genes expression. IDT online software (https://sg.idtdna.com/PrimerQuest/Home/Index) was used to design genes specific primers for qRT-PCR.

Gene ID	Primer	Primer sequence(5'~3')
Soff.04G0004130-3G	q-PAL-F/R	F：AGAAGATTTGGGTCGTGTTGG<br>R：TCCTCGGACCTGTTGATCTT
novel.4754	q-ABA-F/R	F：GTTTGTGTCCGTTCTGGATTTG<br>R：GCGGACCTTCTCACTCTTATG
Soff.02G0015160-6G	q-PR1a-F/R	F：CGGGAAGGCCATCTACATAGT<br>R：CAAGTTGAAGAGGTGGGTGTTG
Soff.02G0015160-2C	q-PR1b-F/R	F：GGCTAAGAAGGCCTGTGATTT<br>R：CCAGATGGAGCAACACCAATA
Soff.04G0005200-4F	q-PR1c-F/R	F：GTGGATCTGGTCGGAAATACAA<br>R：CTCACCACCCTTCCCAATAAC
Soff.04G0021370-3D	q-ICS-F/R	F：GTTGACTGTTTGCGAGGTTTG<br>R：CCCTTGTTCTGCCTTCTCTATG
novel.18733	q-NPR1a-F/R	F：CCGTGGTCCATGTTCTACATTA<br>R：CTCGAGATCGACCTCCAAATTC
novel.23616	q-NPR1b-F/R	F：CCGTGGTCCATGTTCTACATTA<br>R：CTCGAGATCGACCTCCAAATTC
GAPDH	GAPDH	F：AAGGGTGGTGCCAAGAAGG<br>R：CAAGGGGAGCAAGGCAGTT
